# Twelve Years' Experience with Direct-to-Consumer Advertising of Prescription Drugs in Canada: A Cautionary Tale

**DOI:** 10.1371/journal.pone.0005699

**Published:** 2009-05-27

**Authors:** Barbara Mintzes, Steve Morgan, James M. Wright

**Affiliations:** 1 Department of Anesthesiology, Pharmacology and Therapeutics, University of British Columbia, Vancouver, Canada; 2 School of Population and Public Health and Centre for Health Services and Policy Research, University of British Columbia, Vancouver, Canada; 3 Departments of Anesthesiology, Pharmacology and Therapeutics and Medicine, University of British Columbia, Vancouver, Canada; Mayo Clinic, United States of America

## Abstract

**Background:**

Direct-to-consumer advertising (DTCA) of prescription drugs is illegal in Canada as a health protection measure, but is permitted in the United States. However, in 2000, Canadian policy was changed to allow ‘reminder’ advertising of prescription drugs. This is a form of advertising that states the brand name without health claims. ‘Reminder’ advertising is prohibited in the US for drugs that have ‘black box’ warnings of serious risks. This study examines spending on DTCA in Canada from 1995 to 2006, 12 years spanning this policy shift. We ask how annual per capita spending compares to that in the US, and whether drugs with Canadian or US regulatory safety warnings are advertised to the Canadian public in reminder advertising.

**Methodology/Principal Findings:**

Prescription drug advertising spending data were extracted from a data set on health sector spending in Canada obtained from a market research company, TNS Media Inc. Spending was adjusted for inflation and compared with US spending. Inflation-adjusted spending on branded DTCA in Canada grew from under CAD$2 million per year before 1999 to over $22 million in 2006. The major growth was in broadcast advertising, accounting for 83% of spending in 2006. US annual per capita spending was on average 24 times Canadian levels. Celebrex (celecoxib), which has a US black box and was subject to three safety advisories in Canada, was the most heavily advertised drug on Canadian television in 2005 and 2006. Of 8 brands with >$500,000 spending, which together accounted for 59% of branded DTCA in all media, 6 were subject to Canadian safety advisories, and 4 had US black box warnings.

**Conclusions/Significance:**

Branded ‘reminder’ advertising has grown rapidly in Canada since 2000, mainly due to a growth in television advertising. Although DTCA spending per capita is much lower in Canada than in the US, there is no evidence of safer content or product choice; many heavily-advertised drugs in Canada have been subject to safety advisories. For governments searching for compromise solutions to industry pressure for expanded advertising, Canada's experience stands as a stark warning.

## Introduction

Similarly to all industrialized countries except the United States (US) and New Zealand, Canada prohibits direct-to-consumer advertising (DTCA) of prescription drugs. However, Canada differs from most other countries that prohibit DTCA in that there is considerable population exposure to this advertising in US media. Around 30% of English-speaking Canadians' television viewing is of US satellite and cable TV, which carries DTCA that is illegal in Canada [1].

Canada has experienced pressure for legislative change to introduce DTCA since the mid 1990's. For example, Merck Frosst argued in a 1996 submission to Health Canada that the industry had a legal right to advertise under freedom of expression provisions [2].

The Canadian government has hosted several national consultations on DTCA and introduced two major shifts in administrative policy. First, a 1996 Health Canada advertising policy statement [3] redefined the boundary between ‘information dissemination’ and ‘advertising.’ The redefinition appears to have provided tacit government approval for unbranded ‘disease-oriented’ advertisements [4]. These advertisements mention a condition and suggest viewers or readers ‘ask your doctor’ about available treatments but do not mention any brands [5].

Second, in November 2000, Health Canada published an administrative policy paper that allowed branded ‘reminder advertisements’ targeting the general public [6]. A reminder ad is a form of DTCA that states a brand name but does not mention the product's indication or make health claims. The November 2000 policy paper cited a 1975 regulatory amendment [7] (Food & Drugs Act, C.01.044) that was introduced to allow advertising of drug prices and, as described by Health Canada in 1984, thereby “to facilitate comparative shopping” [8].

Branded reminder ads rarely if ever state a product's price. However, Health Canada judged reminder ads to be legal under the price advertising provision because advertising to the public of ‘name, price and quantity’ is allowed. This regulatory approach is unique: Canada is the only country that prohibits DTCA yet makes an exception for branded reminder advertising. Reminder advertisements are prohibited in all other developed countries that ban DTCA. Moreover, although in general the US allows prescription drug advertising to the public, the US FDA imposes restrictions on reminder advertising. These restrictions apply both to ads targeting the public and professionals: no reminder ads are allowed for drugs with a ‘black box’ warning — the strongest US regulatory warning of serious harmful effects [5]. The US restrictions apply both to products within a class with a boxed warning extending to all members of the class (e.g. non-steroidal anti-inflammatory drugs and the risk of gastro-intestinal bleeding), and to product-specific warnings. The rationale for this prohibition is public safety, as reminder advertising fails to provide information on product risks. Canada does not impose analogous limitations on reminder advertising, and also does not have a system of ‘black box’ warnings. However, Health Canada sends out safety advisories to the public and health professionals when new evidence of product risks emerges post-approval.

### Aims of this study

There is no published research, beyond anecdotal reports, on the experience with DTCA in Canada since the administrative policy changes in 1996 and 2000. We therefore aimed to describe annual spending on branded and unbranded advertising by prescription drug manufacturers in Canada from 1995 to 2006, and to compare spending over this period to US DTCA spending. This 12-year period was chosen to span Health Canada's policy changes and, in particular, to provide a time period before and after the year 2000 policy shift regarding branded advertising.

In addition to looking at overall levels and trends in spending on Canadian DTCA, we focused on heavily advertised products in terms of conditions treated and whether or not these products had been subject to regulatory warnings of serious risks, including US ‘black box’ warnings or Health Canada safety advisories.

## Methods

We obtained data from a market research company, TNS Media Inc., which tracks advertising spending in the US and internationally. Data were obtained covering all health sector spending in Canada on television, radio, magazines, newspapers and outdoor billboards for a 12-year period, from 1995 to 2006 (n = 12,372 entries) Data were also obtained from TNS Media on US DTCA spending, with all media combined, from 1997 to 2005. We used published US data on DTCA spending for 1995 [9] and 1996 [10] and IMS Health data for 2006 [11]. These three sources all report on data obtained from TNS Media or Competitive Media Reporting (a company that was bought by TNS Media in 2000). The US data cover all types of DTCA: full product advertising (with both brand names and health claims), reminder advertising, and unbranded ‘help-seeking’ ads.

Spending on prescription drug advertising was extracted manually by product and manufacturer name. All brand names were checked against Health Canada's Drug Product Database so that vaccines, over-the-counter drugs and medical devices could be excluded from our analysis. We also excluded brands that are available as both over-the-counter and prescription-only formulations (e.g. Zantac).

Advertisements were classified as ‘unbranded prescription drug advertising’ if the advertiser was a pharmaceutical company that sells prescription-only drugs in Canada and no brand name was mentioned. This includes both corporate image advertising and condition-related entries. An example of the latter type of entry is ‘acid reflux information’ with Astra Zeneca listed as the advertiser.

Annual spending for 1995 to 2005 was adjusted for inflation and converted into year 2006 Canadian dollars using the within country Consumer Price Index (all items). US figures were converted to Canadian dollars, using year-2006 Purchasing Power Parity (general GDP PPP).

We obtained a list of US drugs with black box warnings from a dedicated website, http://formularyproductions.com/blackbox/, and checked the labels of all identified products on the US FDA website's search engine, drugs@fda. Health Canada's safety advisories were obtained from an e-mail subscription service (MedEffect e-Notice) and confirmed on Health Canada's website: http://www.hc-sc.gc.ca/dhp-mps/medeff/advisories-avis/index-eng.php.

## Results

**Figure 1:**
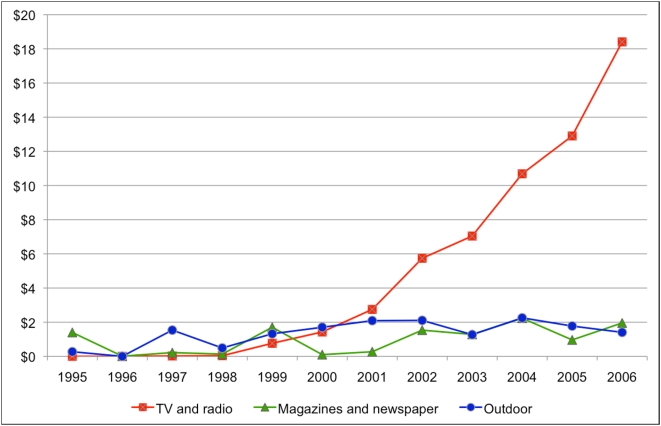
Inflation-adjusted spending on branded direct-to-consumer advertising, 1995 to 2006 (year-2006, CAD$ millions).

Figure 1 presents an overview of inflation-adjusted spending on branded advertising in outdoor, print and broadcast media. Total inflation-adjusted spending on branded DTCA in Canada grew from under $2 million per year prior to 1999 to over $22 million in 2006. The major growth in spending in branded advertising has been in broadcast media, reaching 83% ($18.4 million) of spending in 2006.

Most of the spending on broadcast advertising has been on television ads ($15.5 million in 2006, or 84% of broadcast spending). There was no television DTCA from 1995 to 1997, and television represented only 5% of branded advertising spending in 1998 and 19% in 1999. However, branded television advertising became prominent from 2000 onwards, ranging from 34% to 70% of total branded advertising spending.

From 1995 to 2006, the pharmaceutical industry spent $98.75 million on unbranded pharmaceutical advertising in Canada — see Table 1. From 1995 to 2000, spending on unbranded ads in Canada was three or more times the spending on brand-specific ads. From 2002 onwards, annual spending on branded reminder ads in Canada was consistently higher than on unbranded ads.

**Table 1:** Inflation-adjusted spending on DTCA in the U.S. and Canada, all media, 1995 to 2006 (CAD$ millions)*.

Year	CAD# DTCA brands	CAD spending Branded DTCA	CAD spending Unbranded DTCA†	CAD Total DTCA	USA** Total DTCA	CAD Per capita Branded	CAD per capita Total	USA** per capita Total	Ratio USA/CAD per capita
1995	1	$1.62	$8.73	$10.47	$439.87	$0.06	$0.36	$1.65	5∶1
1996	1	$0.01	$5.40	$5.40	$1,214.85	$0.00	$0.18	$4.51	25∶1
1997	3	$1.58	$8.61	$10.18	$1,138.65	$0.05	$0.34	$4.18	18∶1
1998	6	$0.73	$4.87	$5.60	$1,725.86	$0.02	$0.19	$6.26	38∶1
1999	7	$3.71	$5.39	$9.10	$2,360.05	$0.12	$0.30	$8.46	32∶1
2000	7	$3.22	$9.54	$12.76	$3,262.57	$0.10	$0.42	$11.56	30∶1
2001	7	$5.34	$10.91	$16.14	$3,506.04	$0.17	$0.52	$12.30	25∶1
2002	11	$9.56	$8.32	$17.88	$3,533.65	$0.30	$0.57	$12.27	22∶1
2003	13	$9.69	$5.66	$15.35	$4,303.20	$0.31	$0.48	$14.80	31∶1
2004	19	$17.01	$8.24	$25.25	$5,306.51	$0.53	$0.79	$18.07	22∶1
2005	16	$17.81	$8.29	$26.10	$5,220.93	$0.55	$0.81	$17.61	22∶1
2006	20	$22.30	$14.80	$37.00	$5,829.63	$0.68	$1.14	$19.48	17∶1
Total	48	$92.58	$98.75	$191.23	$37,841.81	-	-	-	-

CAD = Canada; USA = United States.

* inflation-adjusted spending expressed in equivalent of year-2006 dollars.

† includes both unbranded disease-oriented advertising and corporate image advertisements.

** US data converted to CAD$, using year-2006 Purchasing Power Parity (general GDP PPP).

References, US data: 1995: Rosenthal et al. 2002 [8], calculated from Figure 1; 1996: Donohue et al. 2007 [9]; 2006: IMS Health. Total US promotional spend by type, 2007. www.imshealth.com/deployedfiles/imshealth/Global/Content/StaticFile/Top_Line_Data/PromotionalSpendChartWebsite.pdf.

All other Canadian and US data from TNS Media Inc.

A total of CAD$191.23 million was spent on branded and unbranded DTCA in Canada between 1995 and 2006. Over the same period, CAD$36.19 billion was spent on DTCA in the US. Even on a per capita basis, DTCA spending in Canada was much lower than in the US during the entire time period. However, in relative terms DTCA spending in Canada has grown more rapidly since 2001 than DTCA spending in the US. Spending in 2006 was over double the amount spent in 2001; whereas US spending increased by 66% over the same time period.

DTCA spending in Canada is highly concentrated on relatively few products, particularly early in the period being analyzed. Only one product per year was advertised in 1995 and 1996; this grew to 7 products in 1999, 13 in 2003 and 20 in 2006. In total, 48 brands were advertised to the public over the 12-year period. (Table 1)

### Advertised products and conditions

Table 2 lists the top 15 products by advertising spending from 2001 to 2006, representing 99% of spending within this time period, and 95% of spending from 1995 to 2006. Viagra (sildenafil) tops the list and is responsible for 26% of spending from 2001 to 2006.

**Table 2:** Top 15 brands by advertising spending, all media, 2001–2006.

Product	Manufacturer	Indication	CAD$ millions	% total DTCA spend
Viagra (sildenafil)	Pfizer	Impotence	$21.19	26%
Botox (botulinum toxin)	Allergan	Cosmetic	$10.71	13%
Alesse (LNG/EE)	Wyeth Ayerst	Contraception	$8.46	10%
Lipitor (atorvastatin)	Pfizer	Lipid lowering	$8.12	10%
Cialis (tadalafil)	Eli Lilly	Impotence	$7.02	9%
Celebrex (celecoxib)	Pfizer	Arthritis	$6.90	8%
Levitra (vardenafil)	Bayer	Impotence	$5.45	7%
Diane-35 (cyproterone/EE)	Berlex/Bayer*	Acne	$3.49	4%
Evra (norelgestromin/EE)	Janssen Ortho	Contraception	$3.21	4%
Zyban (bupropion)	GSK	Smoking cessation	$2.47	3%
Tri Cyclen Lo (norgestimate/EE)	Janssen Ortho	Contraception	$1.07	1%
Valtrex (vancyclovir)	GSK	Herpes	$0.82	1%
Accutane (isotretinoin)	Hoffman-LaRoche	Acne	$0.69	1%
Paxil (paroxetine)	GSK	Depression	$0.52	1%
Nuvaring (etonogestrel)	Organon	Contraception	$0.51	1%
**Total - top 15 brands**			**$80.63**	**99%**
**Total – all brands**			**$81.71**	**100%**

LNG = levonorgestrel; EE = ethinylestradiol.

* Bayer acquired Berlex in 2007.

These advertised products are also concentrated within relatively few indications: 9 of the 15 are contraceptives, impotence or acne treatments. Pfizer is responsible for 44% of spending, on three products, from 2001 to 2006. In 2006, Celebrex (celecoxib) was the most heavily advertised medicine (CAD$6.90 million).

### Products with US black-box warnings

Table 3 lists all of the medicines advertised on television during 2005 and 2006. Advertising was concentrated on eight brands for which advertising spending exceeded CAD$500,000 during either 2005, 2006, or both of these years. In contrast, spending on each of the remaining 11 ‘minimally advertised’ brands was less than $30,000 per year.

**Table 3:** Safety advisories and black box warnings: products advertised on television, 2005 and 2006.

Intensely advertised (annual spending >$500,000)	2005 (% TV spend)	2006 (% TV spend)	Health Canada safety advisory?	FDA black box?
Celebrex (celecoxib)	-	44%	Gastrointestinal risks (2002); Cardiovascular risks (2004; 2005)	Yes
Viagra (sildenafil)	26%	25%	Visual adverse effects (2005; 2006)	No
Cialis (tadalafil)	23%	15%	Visual adverse effects (2005; 2006)	No
Alesse (LNG/EE)	12%	6%	No	Yes (class)
Lipitor (atorvastatin)	-	5%	Counterfeit products (2006)	No
Tri Cyclen Lo / Tri Cyclen (norgestimate/EE)	-	5%	No	Yes (class)
Evra (norelgestromin/estradiol)	26%	-	High estrogen dose; Venous thromboembolism (2005; 2006)	Yes (class)
Diane 35 (cyproterone/estradiol	14%	-	Venous thromboembolism	N/A; not approved
**Minimally advertised (annual spending <$30,000)**				
Enbrel (etanercept)	-	<1%	Serious infections, hepatitis (2006)	Yes
Aricept (donepezil)	-	<1%	No	No
Imitrex (sumatriptan)	-	<1%	No	No
Valtrex (vancyclovir)	-	<1%	No	No
Advair (salmeterol / fluticasone	-	<1%	Asthma mortality – salmeterol (2003; 2005)	Yes
Vesicare (solifenacin)	-	<1%	No	No
Loestrin (norethindrate/estradiol)	-	<1%	No	Yes (class)
Botox (botulonim toxin)	-	<1%	CADR newsletter article	No
Coreg (CARVEDILOL)	-	<1%	Packaging problem, mixed with another product (2005)	-
Accutane (isotretinoin)	<1%	-	Teratogenic effects	Yes
Levitra (vardenafil)	<1%	-	Visual effects (2005; 2006)	No

Seven of the eight brands heavily advertised on television in Canada during 2005 or 2006 are approved for sale in both countries, and four (57%) have US black box warnings. Together these eight brands represent 99.7% of television advertising and 59.2% of total branded DTCA spending over these two years. In three cases, the warnings are for risks shared by the entire drug class: cardiovascular risks associated with use of estrogen-containing contraceptives in women who smoke and are over 35.

### Health Canada warnings

In total, five of the eight heavily advertised products in 2005 and 2006 were subject to Health Canada safety advisories, excluding a warning about counterfeiting of atorvastatin (Lipitor). In addition to celecoxib and the contraceptive patch, Health Canada also sent out a joint warning of visual adverse effects for three erectile dysfunction drugs in the same class: sildenafil, vardenafil and tadalifil [12]. Another product, Diane-35 (cyproterone and estradiol), which is not approved in the US, has been subject to two safety advisories in Canada [13], [14]. It is indicated in Canada as a second line treatment for severe acne in women.

## Discussion

Although Canada's Food & Drugs Act clearly states that advertising of prescription-only drugs to the public is prohibited, the pharmaceutical industry has spent over CAD $90 million on branded advertising in Canada from 1995 to 2006. Almost all (88%) of this spending on branded advertising occurred after Health Canada stated in 2000 that branded reminder advertising was consistent with a regulatory amendment created to encourage price competition in the 1970s. This interpretation in effect created a regulatory loophole allowing reminder advertising to flourish.

The growth in advertising spending since the year 2000 strongly suggests that policy decisions regarding Canada's regulatory provisions matter. Advertisers may not have been as willing to spend the large sums required to produce broadcast (particularly television) ads if Health Canada's policy statements had not provided some assurance that government would allow branded reminder ads to run in Canada.

The safety profile of the products that have been heavily advertised raises a further note of caution. Many of the drugs featured in reminder advertising have been subject to Canadian safety advisories and to US ‘black box’ warnings.

The most heavily advertised product in Canada during 2006 was Celebrex (celecoxib). Celecoxib is a cox-2 selective inhibitor. Similarly to rofecoxib, celecoxib is associated with increased cardiovascular risks in a dose-related manner [15]. Health Canada issued its first safety advisory on celecoxib in 2002 [16], warning physicians of similar risks of gastrointestinal bleeding to other non-steroidal anti-inflammatory drugs. A 2004 advisory focused on cardiovascular risks [17], and in 2005, Health Canada warned physicians not to prescribe this drug to patients with heart disease and recommended restricting prescriptions to : “… the lowest possible dose, and for the shortest, necessary period of time” [18]. The heaviest advertising spending in Canada on celecoxib was in 2006, after this advisory. Celecoxib was also advertised to the US public during 2006.

In 2005, Janssen-Ortho spent CAD $2.1 million advertising the contraceptive patch Evra (norelgestromin/ethinyl estradiol) to the Canadian public. Evra's US black box warning is a class warning for all estrogen-containing contraceptives, but the patch has also been found to have a higher dose of estrogen than expected, leading to increased risks of venous thromboembolism. The FDA has sent out an advisory and required a labeling change as a result [19]. Excess risks of venous thromboembolism also spurred Health Canada to send out two safety advisories warning physicians not to prescribe Diane-35 (cyproterone/ethinyl estradiol) for contraception or mild acne [13], [14]. Although this product is only approved as a second-line treatment for severe acne, it has been widely prescribed for unapproved uses: 45.5% of women in British Columbia who obtained initial prescriptions from1998 to 2003 had no evidence of acne diagnosis or treatment within the previous year [20]. Health Canada judged advertisements for Diane-35 to be illegal, but found it difficult to prevent repeat violations [8].

These examples highlight the disconnect between marketing decisions to run DTCA campaigns aiming to stimulate sales and regulatory warnings attempting to limit use. Topol faulted the US FDA for allowing intensive DTCA for Vioxx (rofecoxib) despite mounting evidence of cardiovascular toxicity [21]. Our analysis indicates, similarly, that regulators in Canada have failed to prevent advertising of products with a serious potential for harm.

The US industry association, PhRMA, announced self-regulatory guidelines in July 2005, prohibiting television reminder advertising [22]. Coming six months after rofecoxib's withdrawal, these guidelines have been interpreted as a response to the safety concerns raised about the effects of DTCA following rofecoxib's withdrawal [23]. There are no published evaluations of the impact of these guidelines in the US. In Canada, spending on televised reminder ads increased in 2006. All of the manufacturers with spending over $500,000 are Canadian subsidiaries of PhRMA members or, in one case, the Canadian subsidiary of a European company with a US subsidiary that is a PhRMA member.

Despite the rise in spending in Canada during recent years, the volume of advertising pales in comparison with the US. US advertisers spent on average 24 times the amount spent per capita in Canada: a total of CAD $36.187 billion from 1995 to 2006. Additionally, although per capita spending is increasing in Canada, annual growth is much lower in absolute terms than in the US: on average CAD $0.12 per year from 2001 to 2006, versus CAD $1.53 in the US. Thus if current trends in both countries continue unchanged, exposure levels would be expected to remain much lower than in the US.

In the US, full product ads are the most common form of televised DTCA [24], [25]. Because of their extra length, they are more expensive than reminder ads. If companies choose this form of advertising for brands that can be legally advertised through reminder ads, it is likely because of a stronger observed effect on sales.

Donohue and colleagues reviewed the experience with US DTCA from 1996 to 2005 [10]. In addition to the higher spending levels, a much broader range of products has been advertised to the public in the US than in Canada over this period. The drug classes with over 30% of promotional spending dedicated to DTCA included statins, proton pump inhibitors and erythropoietin products. The latter are used to avoid the need for transfusions in cancer patients undergoing chemotherapy. A US Congressional hearing critiqued unsubstantiated claims of reduced fatigue and improved quality of life in DTCA promoting these agents for chemotherapy patients [26]. The US FDA issued a black box warning for the class in 2007 of increased mortality, serious cardiovascular and thromboembolic risks, and tumour progression or recurrence, particularly when used in patients with haemoglobin levels over 12 g/dL [27]. The experience with erythropoietin illustrates a key concern about the effects of DTCA on public safety. Many prescription medicines are potentially hazardous and must be used judiciously in order to ensure that for a specific patient, the potential for benefit outweighs the probability of harm. This need for limited use is at odds with advertisers' imperative to stimulate expanded sales.

Despite the legal requirement for risk information in US full product advertising, provision is often inadequate. Minimization or omission of risks is the most frequent US regulatory violation, repeat violations are common, and as DTCA volume has increased over time, the proportion of ads the FDA is able to review has decreased [28].

There is also evidence of poor communication of harmful effects in advertising that meets regulatory requirements. Adults with low literacy who were tested for comprehension of information in television ads scored much lower on risks than benefits [29]. In a systematic sample of magazine ads for HIV/AIDS drugs, 55% of drugs with black box warnings or life-threatening harmful effects provided incomplete information on these risks, and 48% failed to highlight them graphically [30]. Content analyses of systematic samples of DTCA have found that most ads fail to provide the information needed for shared informed treatment choice [31], benefits are described in vague, emotive terms [32] and emotional appeals such as happiness, control over one's life and social approval are common [24].

In sum, the US experience illustrates why allowing full product advertising is not a solution to Canada's problem of reminder advertising for drugs with serious risks. Neither the inadequate communication of risks nor the negative consequences of stimulating use of products with a serious potential for harm would be resolved. From a public health perspective, a better approach would be to address the problem directly, by closing the regulatory loophole that has allowed this advertising to flourish.

This study has several limitations. Our results are purely descriptive. Advertising spending is only a rough proxy for population exposure, and the relationship between spending and exposure varies over time and by media type. We report only on total Canadian spending and could not examine whether advertising intensity differed by province, as might have occurred in response to differences in provincial formulary listings for some advertised drugs. Additionally, as all publicly reported US data on DTCA spending derive from TNS Media, we could not check accuracy against another source. It was not always possible to distinguish corporate image advertisements from unbranded ‘disease-oriented’ ads; spending on unbranded DTCA is therefore likely to be an overestimate. Additionally, although the US restricts reminder advertising of drugs with black box warnings on public health grounds, there has been no evaluation of the health effects of this restriction.

### Conclusions

This review of 12 years of advertising spending in Canada is a sobering reality check: many of the most heavily advertised products have been subject to regulatory warnings of serious risks. If public health is to be taken seriously, Canada's government needs to take action to stop reminder advertising. It makes no sense to send out safety advisories telling physicians to prescribe a drug cautiously because of serious risks and then, using a regulatory loophole created to foster price competition, to turn a blind eye to persuasive advertisements that make the same drug look like an effortless key to happiness and good health. The suggestion to ‘ask your doctor’ is no guarantee that the viewer is protected, as doctors often prescribe medicines that patients request although they might not have otherwise chosen to do so [33].

In 2003 and early 2004, Canada's parliamentary health committee held hearings across the country on pharmaceutical policy, including DTCA. The committee highlighted the problem of reminder advertising, stating that: “*any direct-to-consumer advertising, including reminder ads, could contribute to increased or inappropriate drug consumption*” [34]. Since this committee's investigation, spending on DTCA in Canada has more than doubled. The US experience of widespread harm associated with the use of the heavily advertised arthritis drug Vioxx (rofecoxib) [35] has also led to proposals for restrictions on DTCA as public safety measures, such as the Institute of Medicine's recommendation for a 2-year moratorium on advertising of new drugs [36].

The experience in Canada provides a cautionary tale for governments in the European Union and elsewhere who are attempting to juggle industry demands for greater ability to ‘inform’ the public about their medicines with public, professional and parliamentary reluctance to introduce ‘US-style’ prescription drug advertising.
